# 

**Published:** 1862-10

**Authors:** 


### CHICAGO MEDICAL JOURNAL.

**Terms, $2.00 per Annum.**

### CONTENTS OF THE OCTOBER NUMBER.

ORIGINAL COMMUNICATIONS.

| | Page. |
|---|---|
| Report of Surgical Cases Treated by Dr. Geo. K. Amerman, Attending Surgeon to the Chicago City Hospital | 551 |
| Camp Douglas | 555 |
| Malingering. By Tom O. Edwards, M. D., of Chicago | 561 |
| SELECTED. | |
| Conservative Medicine. By Austin Flint, M. D., Prof. of the Principles and Practice of Medicine in the Bellevue Hospital Medical College, N. Y., and in the Long Island Hospital. (Concluded from September number.) | 583 |
| Editorial and Miscellaneous | 594 |

